# Precision mapping: An innovative tool and way forward to shrink the map, better target interventions, and accelerate toward the elimination of schistosomiasis

**DOI:** 10.1371/journal.pntd.0006563

**Published:** 2018-08-02

**Authors:** Louis-Albert Tchuem Tchuenté, J. Russell Stothard, David Rollinson, Jutta Reinhard-Rupp

**Affiliations:** 1 University of Yaoundé I, Yaoundé, Cameroon; 2 Centre for Schistosomiasis and Parasitology, Yaoundé, Cameroon; 3 Department of Parasitology, Liverpool School of Tropical Medicine, Liverpool, United Kingdom; 4 Global Schistosomiasis Alliance, Department of Life Sciences, Natural History Museum, London, United Kingdom; 5 Merck Global Health Institute, Ares Trading SA, Switzerland, Subsidiary of Merck KGaA, Darmstadt, Germany; Swiss Tropical and Public Health Institute, SWITZERLAND

## Introduction

The mainstay of current schistosomiasis control is preventive chemotherapy (PC) with praziquantel, targeted toward school-aged children, based on the disease endemicity within a subset of surveyed schools, which are classified using parasitological prevalence and intensity of infections [1]. However, it is well known that the current conventional mapping design for schistosomiasis has shown several limitations and may lead to several uncertainties and misclassification of some districts and their eligibility for PC. These inaccuracies prevent successful PC coverage of all populations that need treatment and, therefore, jeopardise the achievement of schistosomiasis elimination [2]. Our recent studies have revealed that precision mapping of schistosomiasis is an essential requirement to move from disease control toward interruption of schistosome transmission in sub-Saharan Africa. As schistosomiasis is a focal disease geographically, having a high-resolution map is necessary if current or future interventions are to be targeted accordingly. Precision mapping is defined as conducting sampling at a much finer geographical resolution, potentially examining all schools within every subunit in each implementation unit in order to eliminate the errors caused by missing the focal variation in schistosomiasis prevalence. Indeed, current mapping protocols and sampling frames perform badly when assessing this landscape.

## Limitations and uncertainties of the current mapping design for schistosomiasis

**Fig 1:**
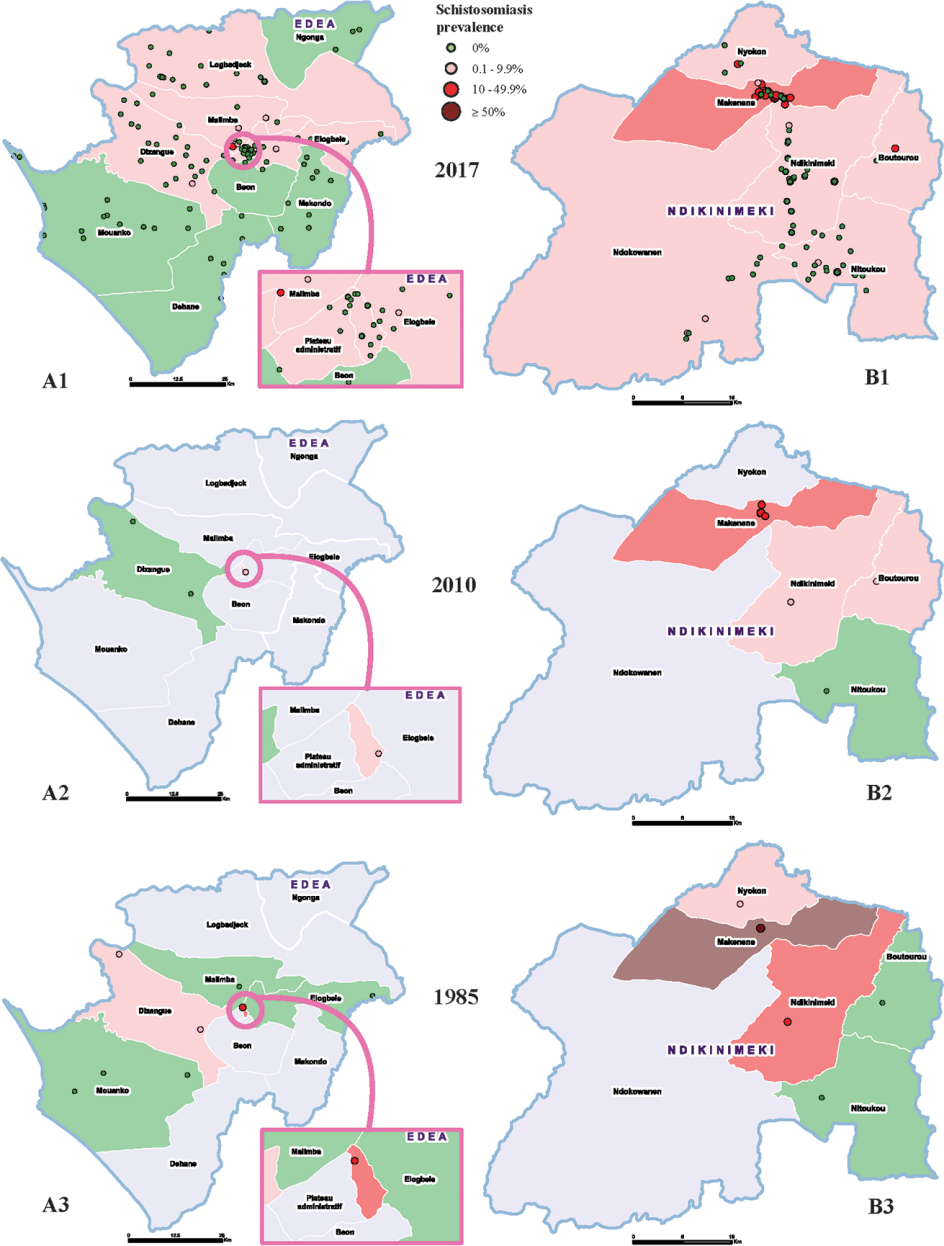
**Comparison of precision and conventional maps of schistosomiasis prevalence in the health districts of Edea (A) and Ndikinimeki (B), Cameroon.** The precision maps (A1 and B1) provide more accurate information on the distribution of schistosomiasis within districts and a clear precision on subdistricts or communities requiring preventive chemotherapy. Differences of subdistrict prevalence between 1985 and 2010 mappings further illustrate the limitations and uncertainties of the conventional mapping (A2 versus A3 and B2 versus B3). Produced with Esri ArcGIS Pro 2.0.

In the current World Health Organization-recommended (WHO) mapping design, in each health district (i.e., the implementation unit), a sub-sample of up to five schools and 50 children per school are selected for parasitological surveys. The eligibility of the district for PC is then determined by the mean prevalence that allows the entire district to be classified as nonendemic (= 0%), low (<10%), moderate (≥10% to <50%), or high risk (≥50%) for schistosomiasis [1]. Although this approach has been suitable in the past, within the context of morbidity control and paucity of drug availability and funding, it is currently not suitable for achieving the schistosomiasis elimination goal and WHO roadmap targets. Indeed, due to the high focality of schistosomiasis transmission and its dependence on several epidemiological and ecological factors (e.g., climatic and physical conditions, type of water bodies, susceptible intermediate snail species and population dynamics, impact of reservoir hosts, intensity of water contacts, etc.) [3], there exists significant difference in infection prevalence between subsettings and schools within the same health districts. The selection of only a few schools to decide for the entire district’s endemicity leads to some uncertainties and errors if the site selection and sampling are not properly undertaken. In this context, a misclassification of districts and the subsequent decision to implement mass PC or not may result in overtreatment in some areas and, most importantly, undertreatment or no treatment in areas that need it most (Fig 1). Examples of the limitations of the current schistosomiasis-mapping design and its impact on the determination of health district eligibility for mass PC in Cameroon have been highlighted in a recent publication by Tchuem Tchuenté and colleagues [2].

## Precision mapping of schistosomiasis

Without clear knowledge of disease transmission foci, countries are not able to adequately plan interventions for schistosomiasis elimination, often requiring sustained long-term efforts. The mapping of disease foci distributions is a critical step in understanding where at-risk populations live, therefore, more accurate mapping is a prerequisite for effective and higher impact control interventions. As schistosomiasis elimination moves forward, there is a need to map more geographical points to deepen the understanding of disease distribution and to guide decision-making. To assess the role precision mapping could play in the stages of moving toward elimination of schistosomiasis, we recently conducted a preliminary study in two health districts in Cameroon, i.e., Edea in the Littoral region, a low-transmission area for the rectal schistosomiasis *Schistosoma guineensis* [4], and Ndikinimeki in the Centre region, where there occurs a high transmission of intestinal schistosomiasis *S*. *mansoni* [5]. Using this new and innovative approach, the study consisted of an exhaustive sampling of all schools (maternal, primary, and secondary) in each of these two districts. A total of 126 and 108 schools and 7,470 and 3,463 school children were sampled in the Edea and Ndikinimeki health districts, respectively. School infection prevalence ranged from 0% to 15% in Edea and 0% up to 50% in Ndikinimeki. The point prevalence of schistosomiasis in all surveyed schools, shown in Fig 1, clearly illustrates the distribution of the disease in each district. The maps show significant variations in schistosomiasis prevalence within health districts and subdistricts. The majority of schools were negative for schistosomiasis. For each district, a comparison of maps obtained using the ‘conventional’ mapping method with those from precision mapping shows significant differences. The precision map illustrates the high focality of schistosomiasis transmission and clearly provides detailed information on high-risk zones and locations where intensified interventions should be focused primarily to obtain higher impact. For example, the health district of Edea is subdivided into 11 health areas, with a total PC-targeted population of 119,157. Interestingly, precision mapping clearly revealed that schistosomiasis occurs in six health areas, and only one health area had an infection prevalence above 10% (the minimum threshold for mass PC for morbidity control). Therefore, the PC-targeted population will be reduced to 12,288 for a morbidity control target or to 91,396, in case the program has the ambition to interrupt the transmission of schistosomiasis. Consequently, the drug needs will be significantly reduced as well. This is the first description of precision mapping of schistosomiasis in Cameroon. However, as the schistosomiasis prevalence may vary significantly from one district to another, the subsequent gains may also vary. Therefore, further studies are required to allow adequate assessment of cost-effectiveness and strategy optimisation of precision mapping.

## Priority interventions for schistosomiasis elimination

On March 22–23, 2017, experts attending the international conference on schistosomiasis in Cameroon, entitled ‘Towards elimination of schistosomiasis: A paradigm shift’, endorsed the precision-mapping approach as one of the priority interventions for schistosomiasis elimination. The conference provided a platform to access all that was new, evolving, challenging, successful, and exciting in schistosomiasis control and elimination. Looking forward to the 2020/2025 WHO roadmap targets and the World Health Assembly WHA65.21 resolution on elimination of schistosomiasis, the conference endorsed the WHO Regional Office for the Africa ‘PHASE approach’, which refers to integrated implementation of a package of PC, health education, access to safe drinking water, sanitation and hygiene, and environmental improvement [6]. The conference put forward four recommendations for priority interventions to move toward schistosomiasis elimination: (i) to expand general access to praziquantel treatment by extending mass PC to preschool-aged children and adults and by increasing the availability of medicines in health centres and treatment stations throughout the year to ensure that all those who seek treatment can receive it; (ii) to complete precision mapping to provide high-resolution information to better focus and tailor PC, to adapt treatment strategies to schistosomiasis transmission dynamics, and, where necessary, to introduce biannual treatment as intensification of current PC campaigns; (iii) to intensify multisectoral actions, which consolidate control and elimination of schistosomiasis that specifically upscale and foster sustainability of PHASE activities; and (iv) to encourage community ownership of the programme with appropriate communication and health education tools that nurture a closer partnership between local and national stakeholders engaged in cross-sectorial actions [7].

## Conclusion

Precision mapping of schistosomiasis gives high-resolution information at the local level. By increasing the map granularity and spatial resolution, precision mapping provides the best evidence-based data to guide intensified interventions in targeted transmission zones and allows for a better and rational utilization of the donated praziquantel and available resources. We therefore believe that it is a promising and innovative tool to shrink the map of schistosomiasis and accelerate the move toward the elimination of schistosomiasis.

## References

[1] World Health Organization. Preventive chemotherapy in human helminthiasis: coordinated use of anthelminthic drugs in control interventions: a manual for health professionals and programme managers Geneva: World Health Organization Press; 2006.

[2] Tchuem Tchuente LA, Rollinson D, Stothard JR, Molyneux D. Moving from control to elimination of schistosomiasis in sub-Saharan Africa: time to change and adapt strategies. Infect Dis Poverty. 2017;6(1):42 10.1186/s40249-017-0256-8 ; PubMed Central PMCID: PMCPMC5319063. 28219412 PMC5319063

[3] Stothard JR, Campbell SJ, Osei-Atweneboana MY, Durant T, Stanton MC, Biritwum NK, et al Towards interruption of schistosomiasis transmission in sub-Saharan Africa: developing an appropriate environmental surveillance framework to guide and to support 'end game' interventions. Infect Dis Poverty. 2017;6(1):10 10.1186/s40249-016-0215-9 ; PubMed Central PMCID: PMCPMC5237522. 28088239 PMC5237522

[4] Tchuem Tchuente LA, Dongmo Noumedem C, Ngassam P, Kenfack CM, Gipwe NF, Dankoni E, et al Mapping of schistosomiasis and soil-transmitted helminthiasis in the regions of Littoral, North-West, South and South-West Cameroon and recommendations for treatment. BMC Infect Dis. 2013;13:602 10.1186/1471-2334-13-602 ; PubMed Central PMCID: PMCPMC3878270. 24365046 PMC3878270

[5] Tchuem Tchuente LA, Kamwa Ngassam RI, Sumo L, Ngassam P, Dongmo Noumedem C, Nzu DD, et al Mapping of schistosomiasis and soil-transmitted helminthiasis in the regions of centre, East and West Cameroon. PLoS Negl Trop Dis. 2012;6(3):e1553 10.1371/journal.pntd.0001553 ; PubMed Central PMCID: PMCPMC3295801. 22413029 PMC3295801

[6] World Health Organization. Regional Strategic Plan for Neglected Tropical Diseases in the African Region 2014–2020. Brazzaville: World Health Organization Regional Office for Africa; 2013.

[7] Ministry of Public Health Cameroon. TES Conference 2017 Report & Recommendations. Towards elimination of schistosomiasis: a paradigm shift. 2017;(22–23 March 2017).

